# Ultrasound‐Guided Liposomal vs. Conventional Bupivacaine Erector Spinae Plane Block Plus Gabapentin for Postherpetic Neuralgia

**DOI:** 10.1002/kjm2.70169

**Published:** 2026-01-12

**Authors:** Xia‐Jing Xu, Li‐Juan Liu, Fa‐Ming Xu, Xiang‐Yu Fang

**Affiliations:** ^1^ Department of Anesthesiology Quzhou Second People's Hospital Quzhou Zhejiang Province China; ^2^ Department of Anesthesiology The Quzhou Affiliated Hospital of Wenzhou Medical University, Quzhou People's Hospital Quzhou Zhejiang Province China

**Keywords:** gabapentin, liposomal bupivacaine, Postherpetic neuralgia, regional anesthesia, ultrasound‐guided erector Spinae plane block

## Abstract

This study aimed to compare efficacy and safety of ultrasound‐guided erector spinae plane block (ESPB) using liposomal (LB) versus conventional bupivacaine (CB), each plus standardized gabapentin, in patients with postherpetic neuralgia (PHN). A total of 116 PHN patients were randomized to ESPB‐LB or ESPB‐CB; both groups received standardized gabapentin. The primary outcome was resting pain assessed by Numeric Rating Scale (NRS). Secondary outcomes were 72‐h event‐free time (time to first rescue medication or resting NRS ≥ 4), cumulative rescue opioid exposure in morphine milligram equivalents (MME), sleep by the Pittsburgh Sleep Quality Index (PSQI), health‐related quality of life by the Patient‐Reported Outcomes Measurement Information System (PROMIS), Patient Global Impression of Change (PGIC), and adverse events (AEs). ESPB‐LB achieved lower resting NRS at day 1, day 3 and day 7 (all *p* < 0.05), with no between‐group differences at day 30 or day 60. Event‐free time favored LB (median 54.10 h vs. 18.95 h; log‐rank *p =* 0.002). LB reduced cumulative MME at 48 h and 72 h, with convergence by days 7–60. PGIC favored LB at days 7 and 30, converging by day 60. Sleep and health‐related quality of life improved similarly with no between‐group differences; AEs were comparable, and no serious events occurred through day 60. In PHN, ESPB‐LB plus gabapentin provided an early advantage characterized by superior early analgesia, higher early responder rates, longer opioid‐free analgesia within 72 h, and short‐term opioid sparing versus ESPB‐CB, while longer‐term outcomes and safety profiles were similar between groups.

## 1 Introduction

Herpes zoster (HZ) arises from reactivation of latent varicella‐zoster virus in cranial or dorsal root ganglia and often produces debilitating dermatomal pain [1]. Its most common chronic complication—postherpetic neuralgia (PHN) ‐ causes persistent neuropathic pain that impairs function and quality of life; reported PHN incidence after HZ ranges from ~5% to > 50% depending on definition and study design, and ~30% of patients may experience pain beyond 1 year [2, 3]. Mechanistically, PHN pain reflects a combination of deafferentation with loss of intraepidermal nerve fibers, “irritable” cutaneous nociceptors, hyperexcitable ectopic pacemaker sites within affected dorsal root ganglia and peripheral nerves, and secondary central sensitization in the spinal dorsal horn [4, 5]. Varicella–zoster virus induces neuropathic changes in DRG, including increased sodium current density and upregulation of Nav1.6/Nav1.7 channels that support ectopic discharges, and behavioral sensitization that is attenuated by gabapentin or sodium‐channel–blocking drugs [6]. Preventive and therapeutic strategies span antivirals, vaccination, corticosteroids, anticonvulsants, and antidepressants [7]. Gabapentin is widely used for PHN but has nonlinear pharmacokinetics, variable absorption, a short half‐life, and dose‐limiting adverse effects (e.g., dizziness and somnolence), challenges that are magnified in older adults with polypharmacy [8].

The erector spinae plane block (ESPB), first described in 2016, has quickly gained favor as a simple, versatile, ultrasound‐guided regional technique with a favorable safety profile across surgical, acute, and chronic pain indications [9]. By depositing local anesthetic deep to the erector spinae muscle at the transverse process, ESPB can spread to dorsal rami, ventral rami, and paravertebral spaces, potentially modulating both somatic afferents and segmental sympathetic fibers that may contribute to sympathetically maintained components of neuropathic pain through sympathetic–sensory coupling in the DRG [10]. For zoster‐related pain, ESPB has emerged as a minimally invasive option: case‐series and observational data suggested meaningful analgesia in refractory PHN [11, 12, 13], and in acute HZ, randomized data showed that ESPB with local anesthetic ± steroid improves pain and quality of life versus standard care [14].

Liposomal bupivacaine (LB; Exparel) provides extended release and is widely used perioperatively; however, its added value over conventional bupivacaine (CB) is context‐dependent. Some studies reported modest reductions in pain or opioid use (e.g., abdominal wall blocks and foot surgery) [15, 16], whereas broader syntheses across peripheral nerve blocks found trivial or no clinically meaningful superiority in pain, opioid consumption, or recovery endpoints [17, 18]. Given that PHN‐associated VZV strains increase Nav1.6/Nav1.7‐mediated sodium currents in sensory neurons‐molecular targets of amide local anesthetics‐prolonged perineural delivery of bupivacaine via a liposomal formulation may more effectively “silence” ectopic DRG firing and dampen early peripheral input during the critical early post‐block window, while entrenched central sensitization likely limits any durable benefit once drug levels wane [5, 6]. In PHN specifically, a randomized double‐blind trial demonstrated that ESPB with 0.25% CB reduced pain during the first week and lowered pregabalin/acetaminophen use through weeks 3–12 versus sham, supporting ESPB with bupivacaine as an effective strategy [14]. Although ESPB‐LB has seen growing clinical use (e.g., lower 0–48 h pain AUC but unchanged opioid in a matched cesarean cohort; favorable postoperative analgesia in a spinal cord stimulator series) [19, 20], rigorous comparative data in PHN are lacking.

Accordingly, we conducted a randomized trial comparing ultrasound‐guided ESPB using liposomal versus conventional bupivacaine, each combined with standardized gabapentin, in thoracic/lumbar PHN. We hypothesized that ESPB‐LB would yield superior early analgesia and short‐term opioid sparing relative to ESPB‐CB with comparable safety, while longer‐term patient‐reported outcomes (sleep, global health, PGIC) would converge between groups.

## 2 Methods and Materials

### 2.1 Ethics Statement

This was a single‐center, prospective, randomized, parallel‐group, assessor‐blinded clinical trial, in accordance with the Declaration of Helsinki. The protocol was approved by the Institutional Review Board of our hospital, and all participants provided written informed consent prior to any study procedures.

### 2.2 Participants

Adults aged 18–75 years with PHN involving the thoracic and/or lumbar dermatomes, defined as neuropathic pain persisting ≥ 3 months after rash onset, were eligible if they had a baseline resting NRS ≥ 4 and the ability to complete all scheduled follow‐ups [21]. Exclusion criteria were known hypersensitivity to any study drugs; coagulopathy or ongoing anticoagulation that contraindicates fascial‐plane injection; active infection at the puncture site; severe spinal deformity or prior surgery that prevents reliable sonographic landmarks; uncontrolled central nervous system disease; significant hepatic impairment (ALT > 50 U/L and/or AST > 45 U/L) or renal impairment (serum creatinine ≥ 1.5 mg/dL); clinically significant cardiac disease; pregnancy or lactation; any invasive pain procedure within the prior month (e.g., paravertebral/epidural injection, radiofrequency ablation, and spinal cord stimulation); abnormal coagulation (INR ≥ 1.5 and/or platelets ≤ 50,000/μL); and any condition judged by investigators to compromise safety or adherence.

### 2.3 Sample Size Calculation

We powered the trial for the primary endpoint (change from baseline in resting NRS) assuming a two‐sided *α* = 0.05, 80% power, and equal allocation. Variability was anchored to contemporary, closely matched data from an RCT in zoster‐associated pain reporting day‐60 pain scores with approximately SDs of ~2.7 (ESPB 2.45 ± 3.05; PVB 2.15 ± 2.70; control 4.30 ± 2.27) [21]. We prespecified a between‐group minimally clinically important difference of 1.5 NRS points (0–10). Under these assumptions, the required sample size was ~51 participants per arm; inflating by ~10% for attrition yielded a target of 58 per arm (total *N* = 116).

### 2.4 Randomization, Allocation Concealment, and Blinding

**FIGURE 1:**
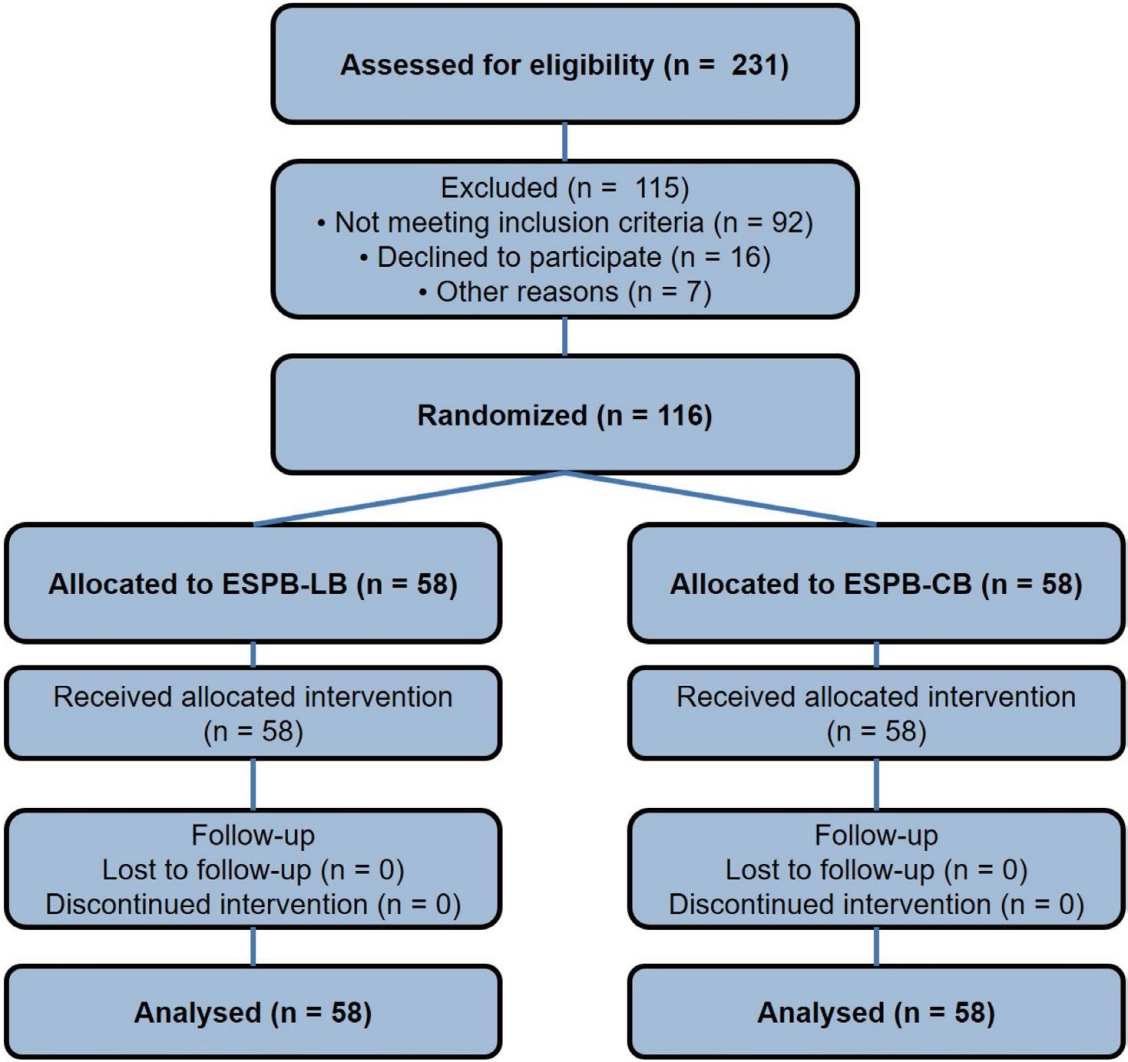
CONSORT study flow and protocol overview.

A total of 116 participants were randomized 1:1 to ESPB‐LB (liposomal bupivacaine) or ESPB‐CB (conventional bupivacaine) using a computer‐generated permuted‐block schedule (block sizes 4–6) prepared by an independent statistician. Allocation was concealed with sequentially numbered, opaque, sealed envelopes. Participants, outcome assessors, data managers, and statisticians were blinded to group assignment. To minimize visual clues from solution appearance, study drugs were prepared by the investigational pharmacy in opaque, identical syringes with equal total volumes and coded labels; participants could not view the syringes. The proceduralist could not be blinded for safety and technical reasons but had no role in outcome assessment or data analysis. The CONSORT flowchart was provided in Figure 1.

### 2.5 Interventions

All participants received a single‐shot, ultrasound‐guided ESPB at baseline by experienced regional anesthesiologists under full asepsis with standard monitoring (ECG, non‐invasive blood pressure, pulse oximetry) in the sitting position. On the affected side, an ultrasound system (Philips CX50) and an 8‐cm echogenic needle were used; laminae were counted to the target level, a 6*–*13 MHz linear probe was placed longitudinally at midline and slid laterally to visualize the transverse process and paraspinal muscles, and an in‐plane cranial‐to‐caudal approach advanced the needle to the dorsal surface of the transverse process. After negative aspiration, the study solution was injected in 1*–*3 mL aliquots into the plane between the transverse process and the anterior fascia of the erector spinae muscle with continuous ultrasound visualization. Patients were randomized to ESPB‐CB (0.25% bupivacaine, total 20 mL) or ESPB‐LB (liposomal bupivacaine [Exparel] 133 mg, 10 mL diluted with 0.9% saline to 20 mL); both were injected incrementally under real‐time ultrasound guidance. Post‐block observation lasted 6 h to monitor for local anesthetic systemic toxicity and procedure‐related adverse events.

All patients also received gabapentin starting at 100 mg three times daily, titrated by 100*–*300 mg every 3*–*7 days (in three divided doses) up to a maximum of 1800 mg/day to achieve effective pain control; after 2 weeks at a stable effective dose, gabapentin was tapered by 100 mg every 3*–*7 days. The rescue analgesic advised to be taken at home was the combination tablet of paracetamol + tramadol, and ondansetron 4 mg was prescribed as needed for nausea.

### 2.6 Follow‐Up and Assessments

Participants were evaluated at baseline (pre‐block) and on days 1, 3, 7, 30, and 60, either in person or by telephone for patient‐reported outcomes. The primary outcome was resting pain intensity on the Numeric Rating Scale (NRS, 0–10; 0 = no pain, 10 = worst imaginable) referencing the prior 24 h [22].

Secondary outcomes included: (1) duration of analgesia—time from block completion to the earlier of first rescue analgesic use or resting NRS ≥ 4 within 72 h; (2) rescue opioid exposure, summarized as morphine milligram equivalents (MME) based on a fixed‐dose combination tablet containing tramadol 37.5 mg plus acetaminophen 325 mg, corresponding to 3.75 MME per tablet (oral tramadol conversion 0.1 MME/mg). Cumulative MME were calculated for prespecified windows (6, 12, 24, 48, and 72 h; days 1–30; days 1–60). Because each rescue tablet contained the same fixed dose of tramadol 37.5 mg + acetaminophen 325 mg, cumulative MME reflects total exposure to this combination. Patients were instructed to take the tramadol–paracetamol tablet only as needed and, at each in‐person or telephone follow‐up, were asked to report the number of tablets taken since the last contact; these self‐reported tablet counts were used to derive cumulative MME; (3) sleep quality by the Pittsburgh Sleep Quality Index (PSQI); seven components scored 0–3, global 0–21, higher = worse sleep [23]; (4) health‐related quality of life by PROMIS Global‐10 (v1.2) with raw responses converted to T‐scores yielding global physical health (GPH) and global mental health (GMH) summary scores, where higher T‐scores indicate better health; additionally, two single PROMIS items were analyzed separately: Global01 (general health, 5‐level; higher = better) and Global09 (ability to carry out social activities/roles, 5‐level; higher = better) [24]; (5) Patient Global Impression of Change (PGIC), a 7‐point global rating of change from 1 (“Very much improved”) to 7 (“Very much worse”) [25]; and (6) adverse events (AEs) and serious adverse events (SAEs) were captured continuously from block to day 60.

### 2.7 Statistical Analysis

Continuous data were inspected for normality with the Shapiro—Wilk test and summarized as mean ± SD or median (IQR), as appropriate. Repeated measures across time were analyzed with a two‐way mixed ANOVA (between‐subjects factor: group; within‐subjects factor: time), including the group × time interaction. When main effects or interactions were significant, Sidak‐adjusted post hoc comparisons were performed. Categorical variables were compared using χ^2^ tests or Fisher's exact tests, as appropriate. Event‐free analgesia within 72 h (time to first rescue or NRS ≥ 4) was analyzed by Kaplan–Meier curves and the log‐rank test. All tests were two‐sided with α = 0.05. Analyses were conducted in GraphPad Prism.

## 3 Results

### 3.1 Baseline Characteristics

There were no statistically significant differences in age, sex distribution, BMI, ASA class, involved dermatomal region (thoracic vs. lumbar), side (right vs. left), number of involved segments (single vs. ≥ 2), PHN duration, presence of mechanical hyperalgesia/allodynia, or prior antiviral therapy during the acute zoster phase (all *p >* 0.05, Table 1). Daily gabapentin doses were comparable between groups across all prespecified time windows. Over days 1–7, the mean daily gabapentin dose was 861 ± 240 mg/day in the ESPB‐LB group and 852 ± 216 mg/day in the ESPB‐CB group (*p =* 0.846). Over days 1–30, doses were 906 ± 198 vs. 869 ± 204 mg/day (*p =* 0.332), and over days 1–60, 850 ± 247 vs. 876 ± 222 mg/day (*p =* 0.550). These findings confirm that background neuropathic pharmacotherapy was balanced between groups, supporting the overall comparability of the two arms prior to intervention. All 116 randomized participants completed the 60‐day follow‐up and were included in the analyses of primary and secondary outcomes.

**TABLE 1:** Baseline characteristics.

Variable	ESPB‐LB (*n* = 58)	ESPB‐CB (*n* = 58)	*p*
Age, years (median [IQR])	56.5 (48–67)	59.5 (52–63.25)	0.428
Female, *n* (%)	28 (48.3)	26 (44.8)	0.861
BMI, kg/m^2^ (mean ± SD)	22.06 ± 1.97	21.48 ± 2.86	0.130
ASA class II/III, *n* (%)	41/17 (70.7/29.3)	42/16 (72.4/27.6)	0.842
Affected dermatomes: thoracic/lumbar, *n* (%)	38/20 (65.5/34.5)	37/21 (63.8/36.2)	0.869
Side: right/left, *n* (%)	31/27 (53.4/46.6)	30/28 (51.7/48.3)	0.872
Number of involved segments: single/≥ 2, *n* (%)	44/14 (75.9/24.1)	45/13 (77.6/22.4)	0.823
PHN duration, months (median [IQR])	4.8 (3.6–6.2)	4.9 (3.5–6.4)	0.903
Mechanical hyperalgesia/allodynia, *n* (%)	45 (77.6)	44 (75.9)	0.845
Prior antiviral therapy in acute HZ, *n* (%)	52 (89.7)	51 (87.9)	0.761
Gabapentin, mg/day (mean ± SD)			
1–7 days	861 ± 240	852 ± 216	0.846
1–30 days	906 ± 198	869 ± 204	0.332
1–60 days	850 ± 247	876 ± 222	0.550

Abbreviations: ASA: American Society of Anesthesiologists physical status; BMI: body mass index; ESPB‐CB: ultrasound‐guided erector spinae plane block with conventional bupivacaine; ESPB‐LB: ultrasound‐guided erector spinae plane block with liposomal bupivacaine; HZ: herpes zoster; IQR: interquartile range; PHN: postherpetic neuralgia.

### 3.2 Early Analgesic Advantage With ESPB‐LB Plus Gabapentin and Later Similarity Between Groups

**FIGURE 2:**
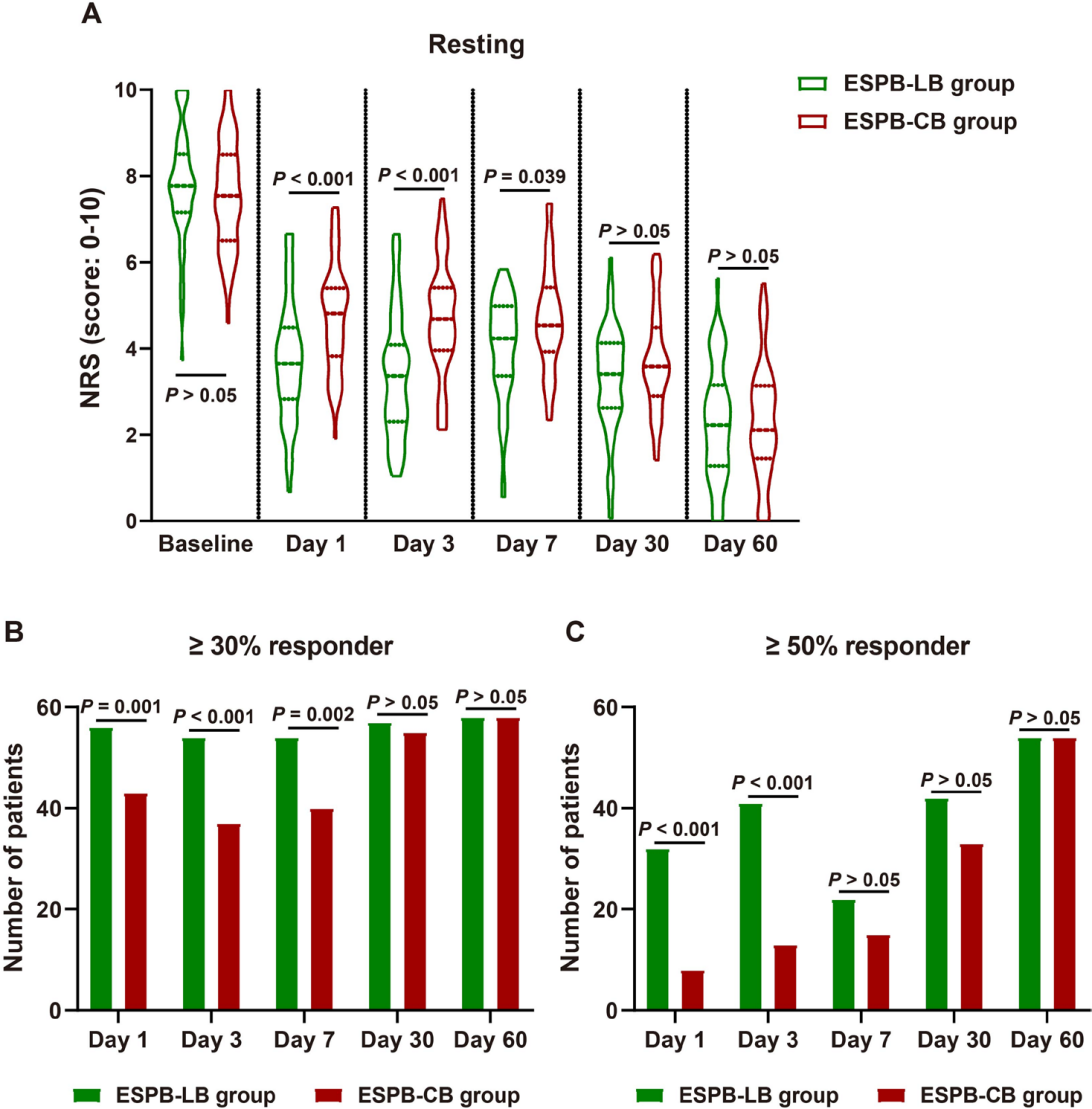
Early analgesic advantage with ESPB‐LB plus gabapentin and convergence over time. (A) Resting NRS (mean ± SD) at baseline, day 1, day 3, day 7, day 30, and day 60 for ESPB‐LB vs. ESPB‐CB. (B) Number of patients achieving ≥ 30% reduction from baseline resting NRS at each time point; (C) Number of patients achieving ≥ 50% reduction.

Compared with ESPB‐CB plus gabapentin, ESPB‐LB plus gabapentin achieved superior early analgesia and higher early responder rates, with no clinically meaningful differences thereafter. Resting NRS scores showed a significant group × time interaction (two‐way ANOVA, interaction *p <* 0.001), with significant main effects of time and treatment group (both *p <* 0.001). At baseline, pain intensity was similarly severe in both groups (ESPB‐LB 7.78 ± 1.34 vs. ESPB‐CB 7.55 ± 1.26; *p >* 0.05). In contrast, ESPB‐LB produced consistently lower resting pain scores during the early post‐block period: on day 1, mean NRS was 3.70 ± 1.40 vs. 4.71 ± 1.23 (*p <* 0.001), and on day 3, 3.38 ± 1.44 vs. 4.70 ± 1.29 (*p <* 0.001). By day 7, the advantage of ESPB‐LB had attenuated but remained statistically significant (4.02 ± 1.19 vs. 4.67 ± 1.19; *p =* 0.039). Thereafter, group differences were no longer evident (both *p >* 0.05): day 30 (3.28 ± 1.24 vs. 3.70 ± 1.20) and day 60 (2.29 ± 1.33 vs. 2.32 ± 1.34) showed similar pain trajectories in both groups, with progressive improvement over time (Figure 2A). Consistently, ESPB‐LB demonstrated a clear early advantage in responder rates (Figure 2B,C): for the ≥ 30% pain reduction threshold, responder rates in the ESPB‐LB group were higher than in the ESPB‐CB group at days 1, 3, and 7 (day 1: 96.6% vs. 74.1%, *p =* 0.001; day 3: 93.1% vs. 63.8%, *p <* 0.001; day 7: 93.1% vs. 69.0%, *p =* 0.001), with both groups approaching ceiling response by days 30 and 60 (≥ 94.8%, *p >* 0.05). For the stricter ≥ 50% responder threshold, ESPB‐LB again showed a clear early advantage (day 1: 55.2% vs. 13.8%, *p <* 0.001; day 3: 70.7% vs. 22.4%, *p <* 0.001), whereas differences at days 7 and 30 did not reach statistical significance (*p =* 0.232 and *p =* 0.120, respectively), and rates converged by day 60 (93.1% in both groups, *p =* 1.000).

### 3.3 Early Analgesic Superiority With Liposomal Bupivacaine Within 72 h

**FIGURE 3:**
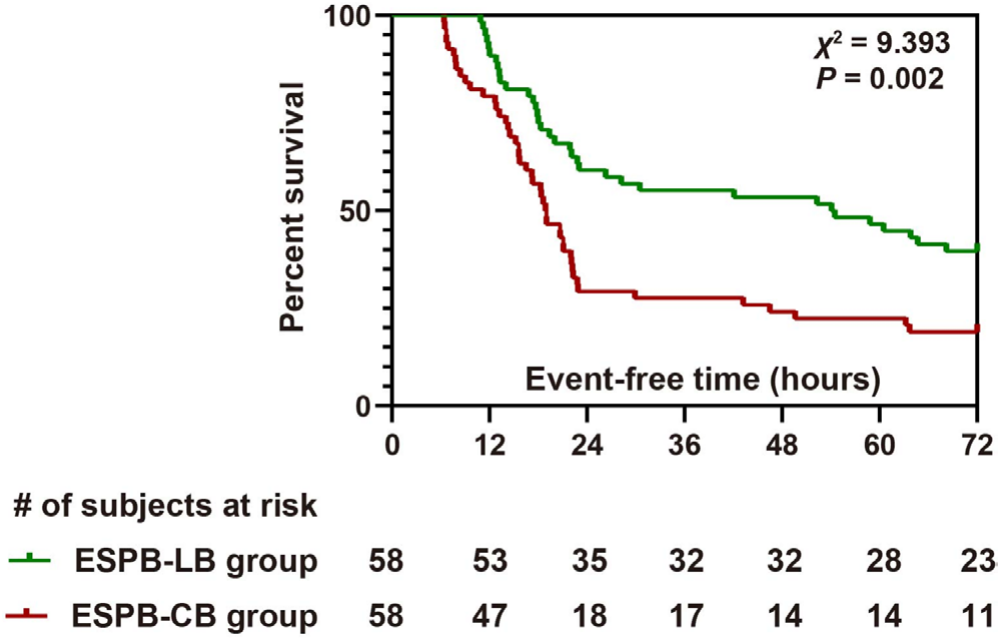
Kaplan—Meier curves for event‐free analgesia within 72 h post‐block. ESPB‐LB exhibited significantly longer event‐free (analgesia) time than ESPB‐CB (log‐rank χ^2^ = 9.393, *p =* 0.002). Median time to event: 54.10 h (LB) versus 18.95 h (CB).

Within the first 72 h after block, ESPB‐LB provided significantly longer event‐free (analgesia) time than ESPB‐CB. The survival curves differed by both the log‐rank test (χ^2^ = 9.393, *p =* 0.002, Figure 3). The median time to event was 54.1 h with LB versus 18.95 h with CB. Event/censoring counts were 35 events/23 censored (LB) and 47 events/11 censored (CB). Consistently, cumulative MME was lower with LB over the same window, reaching significance at 48 h (LB 4.46 ± 3.81 vs. CB 6.66 ± 5.12 MME; *p =* 0.009) and 72 h (LB 6.08 ± 4.54 vs. CB 7.95 ± 5.62 MME; *p =* 0.049, Table 2). By 7, 30, and 60 days, cumulative MME no longer differed between groups (all *p >* 0.05). Within the first 48 and 72 h, the proportion of patients who used at least one dose of rescue tramadol–paracetamol was similar between groups (0–48 h: 42/58 [72.4%] with ESPB‐LB vs. 48/58 [82.8%] with ESPB‐CB, *p =* 0.265; 0–72 h: 47/58 [81.0%] vs. 51/58 [87.9%], *p =* 0.443). By days 7, 30, and 60, all patients in both groups had used rescue analgesia at least once (58/58 [100%] in each group). Thus, early differences in cumulative MME reflect lower rescue requirements in the ESPB‐LB group rather than systematic differences in the proportion of patients using rescue medication. Collectively, LB produced a clear early opioid‐sparing effect and more durable analgesia through 72 h, with rescue‐analgesic use converging by 7–60 days.

**TABLE 2:** Cumulative rescue‐analgesic consumption (MME) by timepoint, mean ± SD.

Timepoint	ESPB‐LB group (*n* = 58)	ESPB‐CB group (*n* = 58)	*p*
Cumulative MME, mean ± SD			
6 h	0.84 ± 1.73	0.97 ± 1.93	0.700
12 h	1.75 ± 2.46	1.94 ± 2.92	0.700
24 h	2.91 ± 3.30	3.75 ± 4.27	0.240
48 h	4.46 ± 3.81	6.66 ± 5.12	0.009
72 h	6.08 ± 4.54	7.95 ± 5.62	0.049
7 days	17.93 ± 5.59	19.59 ± 5.75	0.115
30 days	59.52 ± 11.00	60.57 ± 12.88	0.640
60 days	106.35 ± 16.14	105.11 ± 17.54	0.690
Any rescue opioid, *n* (%)			
48 h	42 (72.4%)	48 (82.8%)	0.265
72 h	47 (81.0%)	51 (87.9%)	0.443
7 days	58 (100.0%)	58 (100.0%)	1.000
30 days	58 (100.0%)	58 (100.0%)	1.000
60 days	58 (100.0%)	58 (100.0%)	1.000

*Note:* Rescue opioid exposure was summarized as morphine milligram equivalents (MME) using a fixed‐dose combination tablet containing tramadol 37.5 mg + acetaminophen 325 mg; only tramadol contributes to MME, with an oral conversion of 0.1 MME per mg (i.e., 3.75 MME per tablet). Cumulative MME were computed over prespecified windows (6, 12, 24, 48, 72 h; Days 1–30; Days 1–60).

### 3.4 Sleep and Global Health Improved Similarly Across Groups Without Between‐Group Differences

Across 60 days, both ESPB‐LB and ESPB‐CB showed comparable improvements in sleep and global health, with no between‐group differences at any timepoint (Table 3). By day 60, PSQI medians decreased from poor‐sleep ranges at baseline to 5.88 (LB) vs. 7.08 (CB) (*p =* 0.967). PROMIS Global Physical Health T‐scores were ~42.3 vs. ~42.3; no between‐group difference, and PROMIS Global Mental Health improved similarly (*p =* 0.942). Patient‐reported global health shifted similarly in both groups (Global01 median 4.00 vs. 4.00, *p =* 0.949; Global09r median 3.50 vs. 4.00, *p =* 0.391). Overall, sleep quality and global health improved over time in both arms without detectable between‐group differences.

**TABLE 3:** Sleep quality and global health over time in ESPB‐LB versus ESPB‐CB groups.

Variable	Timepoint	ESPB‐LB group (*n* = 58)	ESPB‐CB group (*n* = 58)	*p*
PSQI	Baseline	11.27 (9.34–13.09)	10.11 (7.18–13.07)	0.594
	Day 30	6.72 (4.35–8.60)	7.42 (5.01–9.96)	0.851
	Day 60	5.88 (3.84–5.88)	7.08 (4.22–9.50)	0.967
PROMIS Global	**Global physical health**			
	Baseline	32.40 (26.70–34.90)	32.40 (29.60–32.40)	0.993
	Day 30	39.80 (37.40–44.90)	39.80 (37.40–42.30)	0.595
	Day 60	42.30 (39.80–47.70)	42.30 (39.80–44.90)	0.836
	**Global mental health**			
	Baseline	41.10 (36.92–43.50)	41.10 (38.80–43.50)	0.980
	Day 30	48.30 (43.50–50.80)	47.05 (43.50–48.30)	0.390
	Day 60	49.55 (45.80–53.30)	50.80 (45.80–53.30)	0.942
	**Global01 (self‐rated health) response**			
	Baseline	2.00 (2.00–3.00)	2.50 (2.00–3.00)	0.231
	Day 30	3.00 (3.00–4.00)	3.00 (3.00–4.00)	0.790
	Day 60	4.00 (3.00–4.00)	4.00 (3.00–4.00)	0.949
	**Global09r (ability to carry out social activities and roles)**			
	Baseline	3.00 (2.00–3.00)	3.00 (2.00–3.00)	0.112
	Day 30	3.00 (3.00–4.00)	3.00 (3.00–4.00)	0.735
	Day 60	3.50 (3.00–4.00)	4.00 (3.00–4.00)	0.391

*Note:* Pittsburgh Sleep Quality Index (PSQI, 0–21; higher = worse; > 5 commonly indicates poor sleep). Values are median (IQR) unless noted. PROMIS Global Physical Health T‐score and PROMIS Global Mental Health T‐score are standardized (mean 50, SD 10; higher = better). Global01 and Global09r are 5‐point Likert items (5 = Excellent to 1 = Poor). ESPB‐LB: ultrasound‐guided erector spinae plane block with liposomal bupivacaine; ESPB‐CB: ultrasound‐guided erector spinae plane block with conventional bupivacaine.

### 3.5 Comparable 60‐Day Safety Between ESPB‐LB and ESPB‐CB Plus Gabapentin

Over the 60‐day follow‐up, adverse events were generally mild to moderate and occurred with similarly low frequency in both groups (Table 4). The most common opioid/gabapentin‐related events were nausea, constipation, and dizziness, with no significant differences between ESPB‐LB and ESPB‐CB. Overall, 17/58 (29.3%) patients in the ESPB‐CB group and 22/58 (37.9%) in the ESPB‐LB group experienced at least one adverse event (*p =* 0.432). When restricted to the early post‐block period, any opioid/gabapentin‐related adverse event occurred in 12/58 (20.7%) vs. 15/58 (25.9%) patients within 0–48 h and in 15/58 (25.9%) vs. 17/58 (29.3%) within 0–72 h in the ESPB‐CB and ESPB‐LB groups, respectively (all *p >* 0.05). No serious drug‐ or block‐related adverse events (e.g., pneumothorax and local anesthetic systemic toxicity) were observed.

**TABLE 4:** Opioid‐, gabapentin‐, and block‐related adverse events after ESPB [*n* (%)].

Adverse event	ESPB‐CB (*n* = 58)	ESPB‐LB (*n* = 58)	*p*
0–60 days			
Nausea	8 (13.8%)	12 (20.7%)	0.462
Constipation	6 (10.3%)	7 (12.1%)	1.000
Pyrexia (fever)	2 (3.4%)	6 (10.3%)	0.273
Dizziness	5 (8.6%)	6 (10.3%)	1.000
Pruritus	3 (5.2%)	3 (5.2%)	1.000
Transient limb weakness	2 (3.4%)	3 (5.2%)	1.000
Urinary retention	1 (1.7%)	2 (3.4%)	1.000
Local hematoma	1 (1.7%)	1 (1.7%)	1.000
Puncture‐site infection	1 (1.7%)	0 (0.0%)	1.000
Any adverse event (0–60 days)	17 (29.3%)	22 (37.9%)	0.432
Any opioid/gabapentin‐related AE			
0–48 h	12 (20.7%)	15 (25.9%)	0.661
0–72 h	15 (25.9%)	17 (29.3%)	0.836

*Note:* Opioid/gabapentin‐related AEs included nausea, constipation, dizziness, pruritus, and urinary retention. Block‐related AEs included transient limb weakness and local hematoma.

Abbreviations: ESPB‐CB: ultrasound‐guided erector spinae plane block with conventional bupivacaine; ESPB‐LB: ultrasound‐guided erector spinae plane block with liposomal bupivacaine.

### 3.6 Early PGIC Advantage With ESPB‐LB and Narrowing Group Differences by Day 60

**FIGURE 4:**
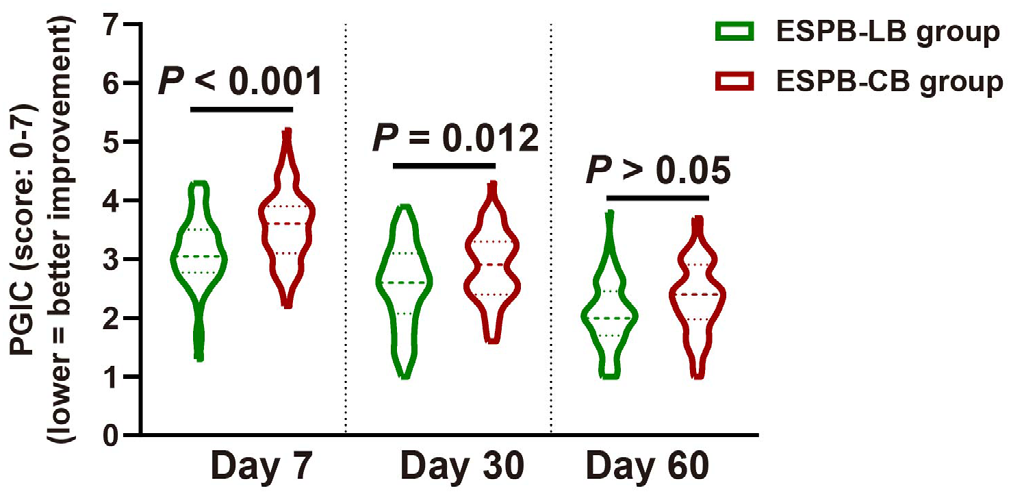
Patient Global Impression of Change (PGIC) over time (days 7, 30, and 60). Lower scores indicate greater improvement (1 = “Very much improved”, 7 = “Very much worse”).

In 116 PHN patients, PGIC showed no group × time interaction (*p =* 0.514) but significant effects of time (*p <* 0.001) and group favoring ESPB‐LB overall (Figure 4): at day 7, ESPB‐LB was 3.10 ± 0.61 vs. ESPB‐CB 3.56 ± 0.62 (*p <* 0.001); at day 30, 2.55 ± 0.70 vs. 2.88 ± 0.60 (*p =* 0.012); by day 60, 2.06 ± 0.58 vs. 2.34 ± 0.61 (*p =* 0.051), indicating greater early global improvement with ESPB‐LB plus gabapentin and similar patient‐perceived status thereafter.

## 4 Discussion

In this randomized trial of thoracic/lumbar PHN, ESPB‐LB plus standardized gabapentin produced a distinct early analgesic advantage over ESPB‐CB, evidenced by lower day‐1, day‐3, and day‐7 resting NRS, higher early responder rates, longer event‐free (analgesia) time through 72 h, and lower cumulative rescue MME at 48–72 h. Thereafter, pain scores, responder rates, MME through 7–60 days, sleep (PSQI), PROMIS global health (GPH/GMH, Global01/Global09), and PGIC converged between groups, and safety was comparable with no serious adverse events.

The longer event‐free time (median 54.10 h vs. 18.95 h), lower early pain scores, and reduced 48–72 h MME with LB are consistent with the extended perineural residence and sustained release of bupivacaine from multivesicular liposomes at the ESP plane, promoting prolonged blockade of dorsal and ventral rami and, by spread, paravertebral/intercostal pathways. Mechanistically, extended local anesthetic availability may dampen early peripheral input and spinal sensitization, thereby delaying rescue needs. Notably, the between‐group difference in mean day‐1 resting NRS—although statistically significant—was approximately 1.0 point and thus represents a modest average improvement below our prespecified 1.5‐point minimally clinically important difference. This modest shift in mean pain scores can nonetheless coexist with a large separation in event‐free time because these metrics capture different dimensions of analgesic benefit: mean NRS compresses the distribution of pain intensities, whereas event‐free time is defined by the interval until either rescue use or NRS ≥ 4 and is therefore highly sensitive to the proportion of patients who remain below a clinically relevant pain threshold without rescue. In our trial, the marked prolongation of event‐free time with LB, together with higher early responder rates and lower early MME, indicates that more patients experienced sustained low pain without needing rescue, even though the average day‐1 NRS difference was numerically modest. In the specific context of chronic PHN, this early “window of advantage” is biologically plausible: acute zoster–related nerve injury and deafferentation generate persistent ectopic discharges in injured primary afferents and drive central sensitization within dorsal horn circuits, so that the spinal cord continues to behave as an amplifier long after rash resolution [4, 26]. Varicella‐zoster virus strains isolated from patients with PHN increase sodium current density and upregulate Nav1.6/Nav1.7 expression in sensory neurons, providing a substrate for DRG hyperexcitability and spontaneous ectopic firing that can be suppressed by local anesthetic blockade only while sufficient perineural drug is present [6, 27]. Our findings align with multiple recent randomized studies reporting that single‐shot LB in thoracic interfascial or paravertebral blocks can improve early recovery (24–48 h), prolong time to first rescue, and in some trials reduce short‐term opioid use and resting pain burden, albeit with heterogeneous effects on total 48‐h opioid use and later pain outcomes, and with safety comparable to CB [28, 29, 30]. In a liver‐resection cohort, LB‐paravertebral reduced 0–72 h opioid use and prolonged time to first opioid, while longer‐term pain and QoR were similar to CB, and safety was comparable despite higher plasma bupivacaine and greater TNF‐α levels postoperatively [31]. Together, these data and our trial support a time‐limited, early advantage of LB in truncal plane/para‐vertebral contexts. This pattern is consistent with pharmacokinetic and clinical data for liposomal bupivacaine: multivesicular liposomes provide prolonged but not indefinite release of bupivacaine over approximately 48–72 h, with an initial rapid distribution phase followed by slower elimination and substantial interindividual variability in block duration [32, 33]. Recent reviews further emphasize that, although perineural LB can modestly extend analgesia compared with plain bupivacaine in some settings, its incremental benefit in terms of pain scores, opioid consumption, and recovery is generally small and highly context‐dependent [34, 35].

Despite early benefits, NRS, responder rates, and cumulative MME converged by day 7–60, and PSQI, PROMIS (GPH/GMH; Global01/Global09), and PGIC did not differ between groups at later time points. Several factors likely explain this pattern: (i) single‐shot ESPB in a chronic neuropathic state yields diminishing perineural drug levels beyond 72 h; (ii) standardized gabapentin titration and uniform rescue algorithms likely homogenized analgesia trajectories; and (iii) PHN pathophysiology (deafferentation, central sensitization) may limit the durable impact of any single perineural exposure once early nociceptive input is curtailed. Taken together, our data suggest that LB primarily extends the period during which abnormal peripheral input from hyperexcitable DRG and injured cutaneous afferents is effectively “silenced,” but does not fundamentally reverse the established central changes that maintain PHN. Once LB concentrations at the ESP plane fall below an effective threshold, ectopic firing and central gain re‐emerge, and longitudinal trajectories become dominated by disease biology and ongoing systemic neuropathic pharmacotherapy rather than by the choice of local anesthetic formulation [34, 36]. Convergence mirrors mixed literature beyond 48–72 h: in shoulder arthroplasty, LB versus CB interscalene blocks showed similar pain and opioid metrics beyond selected early windows and no differences in severe pain or rehospitalization [37, 38]. Likewise, in Wang et al., later pain AUC and sleep at 3 months were similar [31].

Our findings extend PHN‐specific evidence that ESPB with bupivacaine improves early pain and reduces co‐analgesic needs [14, 21] by showing that LB augments the early phase (longer event‐free time and modest 48–72 h opioid sparing) without altering intermediate (7–60 days) patient‐reported outcomes when background neuropathic pharmacotherapy is standardized. From a practical perspective, a single LB‐ESPB therefore appears best suited for scenarios in which rapid early pain control and short‐term opioid minimization are priorities (e.g., severe acute PHN flares or initiation of rehabilitative interventions), whereas meaningful modification of long‐term PHN trajectories likely requires more sustained modulation of segmental input and central sensitization. Case reports and small series describing repeated ESPB sessions or catheter‐based continuous ESPB in PHN have suggested cumulative improvements in pain and function over time [4, 39], supporting the hypothesis that serial or continuous truncal blocks—potentially with LB—could provide a more durable impact on centrally maintained pain than a single extended‐release injection. This supports a pragmatic view: LB may be most valuable when early pain control and opioid minimization are priorities, whereas longer‐term symptom trajectories in PHN appear driven by disease biology and adjunct pharmacotherapy rather than local anesthetic formulation alone. Across 60 days, AE profiles were similar and no serious events occurred, consistent with perioperative data showing comparable safety of LB and CB in peripheral blocks [28, 29, 30, 31]. Although LB can yield higher systemic bupivacaine levels, clinically important toxicity remained absent, aligning with prior reports of a favorable cardiac/CNS safety profile when dosed appropriately.

Several limitations should be acknowledged. First, this was a single‐center trial with a modest sample size, which may limit generalizability. Second, the anesthesiologist performing ESPB could not be blinded to group allocation, which raises the possibility of subtle performance bias in needle manipulation or injectate distribution despite the use of a standardized ultrasound‐guided protocol and blinded outcome assessment. However, block technique, volume, and imaging landmarks were strictly standardized, and all proceduralists were experienced, which we believe mitigates the risk of systematic differences in ESPB quality between groups. Third, we acknowledge that rescue analgesic use was based on patient self‐report rather than direct pill counting, which may introduce recall bias; however, the standardized regimen and similar opioid exposure between groups over 7–60 days make it unlikely that differential adherence explains the observed early analgesic differences. Finally, we evaluated only a single‐shot ESPB regimen; future work should evaluate whether catheter‐based or repeat‐block strategies, potentially in combination with LB, can safely extend segmental analgesia and more effectively target central sensitization in chronic PHN.

In conclusion, in adults with thoracic/lumbar PHN receiving multimodal care, ultrasound‐guided ESPB with LB produced a time‐limited early advantage—lower pain, higher early responder rates, longer event‐free analgesia (0–72 h), and modest opioid sparing at 48–72 h—compared with CB. By 7–60 days, pain, opioid exposure, sleep, global health, and PGIC converged between groups, and safety profiles were comparable with no serious adverse events. Clinically, ESPB‐LB is most useful when early analgesia and short‐term opioid minimization are priorities; durable benefits likely depend on disease biology and ongoing neuropathic pharmacotherapy.

## Funding

This work was supported by Clinical Research Fund Project of Pain Physicians Branch of Zhejiang Medical Doctor Association in 2024 (No. YS2024‐50‐002).

## Conflicts of Interest

The authors declare no conflicts of interest.

## Data Availability

The data that support the findings of this study are available from the corresponding author upon reasonable request.
